# Excessive Supraventricular Ectopic Activity Is Indicative of Paroxysmal Atrial Fibrillation in Patients with Cerebral Ischemia

**DOI:** 10.1371/journal.pone.0067602

**Published:** 2013-06-28

**Authors:** Mark Weber-Krüger, Klaus Gröschel, Meinhard Mende, Joachim Seegers, Rosine Lahno, Beatrice Haase, Cord-Friedrich Niehaus, Frank Edelmann, Gerd Hasenfuß, Rolf Wachter, Raoul Stahrenberg

**Affiliations:** 1 Clinic for Cardiology and Pneumology, University of Göttingen, Göttingen, Germany; 2 Clinic and Polyclinic for Neurology, University of Mainz, Mainz, Germany; 3 Coordination Centre for Clinical Trials, University of Leipzig, Leipzig, Germany; University of Queensland, Australia

## Abstract

**Background:**

Detecting paroxysmal atrial fibrillation (PAF) in patients with cerebral ischemia is challenging. Frequent premature atrial complexes (PAC/h) and the longest supraventricular run on 24-h-Holter (SV-run_24 h_), summarised as excessive supraventricular ectopic activity (ESVEA), may help selecting patients for extended ECG-monitoring, especially in combination with echocardiographic marker LAVI/a’ (left atrial volume index/late diastolic tissue Doppler velocity).

**Methods:**

Retrospective analysis from the prospective monocentric observational trial Find-AF (ISRCTN-46104198). Patients with acute stroke or TIA were enrolled at the University Hospital Göttingen, Germany. Those with sinus rhythm at presentation received 7-day Holter-monitoring. ESVEA was quantified in one 24-hour interval free from PAF. Echocardiographic parameters were assessed prospectively.

**Results:**

PAF was detected in 23/208 patients (11.1%). The median was 4 [IQR 1; 22] for PAC/h and 5 [IQR 0; 9] for SV-run_24 h_. PAF was more prevalent in patients with ESVEA: 19.6% vs. 2.8% for PAC/h >4 vs. ≤4 (p<0.001); 17.0% vs. 4.9% for SV-run_24 h_ >5 vs. ≤5 beats (p = 0.003). Patients with PAF showed more supraventricular ectopic activity: 29 PAC/h [IQR 9; 143] vs. 4 PAC/h [1]; [14] and longest SV-run_24 h_ = 10 [5]; [21] vs. 0 [0; 8] beats (both p<0.001). Both markers discriminated between the PAF- and the Non-PAF-group (area under receiver-operator-characteristics-curve 0.763 [95% CI 0.667; 0.858] and 0.716 [0.600; 0.832]). In multivariate analyses log(PAC/h) and log(SV-run_24 h_) were independently indicative of PAF. In Patients with PAC/h ≤4 and normal LAVI/a’ PAF was excluded, whereas those with PAC/h >4 and abnormal LAVI/a’ showed high PAF-rates.

**Conclusions:**

ESVEA discriminated PAF from non-PAF beyond clinical factors including LAVI/a’ in patients with cerebral ischemia. Normal LAVI/a’+PAC/h ≤4 ruled out PAF, while prevalence was high in those with abnormal LAVI/a’+PAC/h >4.

## Introduction

Though one of the most common causes of ischemic stroke, atrial fibrillation in its paroxysmal form can be challenging to diagnose. Bursts of paroxysmal atrial fibrillation (PAF) can be short, they often occur in clusters [1] and are asymptomatic in over 50% of the cases [2]. Diagnostic yield has been shown to increase with duration of monitoring [3]–[5]. Methods for prolonging ECG-monitoring over wider lengths of time have been developed [6]. Nevertheless, extended diagnostics are time-consuming, expensive and cumbersome for both patients and investigators. Therefore it is essential to identify indicators of underlying PAF in patients with cerebral ischemia to focus extended diagnostics on high-risk patients.

As one potential approach, we have demonstrated that morphologic indicators of structural left atrial remodelling in echocardiography can be useful to rule out the presence of PAF within this collective [7]. As a different approach, biomarkers (e. g. natriuretic peptides) may serve to identify patients with PAF [8].

In addition to structural alterations, atrial fibrillation (AF) is also known to be accompanied by a state of electrical instability of the atrium [9]. There are some studies indicating that premature atrial complexes (PAC) [10]–[13] as well as supraventricular (SV) runs [14]–[15] are associated with prevalent or incident AF.

In a retrospective analysis of data from a prospective observational trial including patients with cerebral ischemia, we evaluated the association of frequent PAC and SV-runs on 24-hour Holter as markers of atrial electric instability with the finding of PAF within a prolonged monitoring spell (7-day Holter-monitoring). We also evaluated previously defined cut-offs of comparable trials by Wallmann et al. [13] and Binici et al. [15]. Once the association was confirmed, we combined these electrocardiographic markers with a recently evaluated sensitive echocardiographic indicator of paroxysmal atrial fibrillation [7], equitable with structural myocardial remodelling (left atrial volume index/late diastolic mitral annular velocity = LAVI/a’) to detect those patients after cerebral ischemia with a high probability of underlying PAF.

## Methods

### Study Design and Ethical Statement

The prospective monocentric observational trial Find-AF (ISRCTN 46104198) complies with the declaration of Helsinki, was performed according to the principles of ICH-GCP and was approved of by the local Ethics Committee at the University of Göttingen. Patients were only included after written and informed consent had been obtained.

### Patients

Patients >18 years with suspected cerebral ischemia presenting at the emergency ward of the Göttingen University Hospital were prospectively included. Details on the cohort, detection of PAF by 7-day Holter-monitoring [5], as well as echocardiographic data to predict the presence of PAF [7] have been previously published. The aim of the study was to detect various factors associated with atrial fibrillation in patients with cerebral ischemia to guide future diagnostic pathways as well as therapeutic approaches. Cardiovascular endpoints were prospectively collected at a clinical follow-up visit after one year.

### ECG Monitoring and Analysis

All patients presenting in sinus rhythm received prolonged ECG-monitoring for up to 7 days. The devices were applied early, i.e. at the emergency ward. The Holter-ECG recordings were analysed using the dedicated analysis-software (CardioDay®, getemed Medizin- und Informationstechnik, Teltow, Germany). The workup of the recorded ECG-data took place in two steps: One 24-hour period was analysed in full detail and was forwarded to the treating physician in a timely manner to represent the standard of care. We arbitrarily chose day four and deviated from this day only if the specific recording was unavailable or of explicitly bad quality. The remaining ECG-material was analysed at a later point in time with a clear focus on detecting PAF. The patient’s general practitioner was contacted only in cases when AF (or another obvious heart rhythm disorder) was detected within the prolonged ECG-spell.

PAF was defined as an absolute arrhythmia >30 s without detectable P-waves and without a more likely differential diagnosis.

We calculated the number of PAC per hour and identified the longest SV-run within 24 hours (SVrun_24 h_) as a semi-quantitative surrogate for electrical instability (or excessive supraventricular ectopic activity = ESVEA) of the atrium. In most cases, we used the recording that had been thoroughly evaluated as part of the clinical routine for these analyses. If this specific recording contained an episode of PAF, we reanalysed the next day free from the arrhythmia.

To determine the number of PAC, all automatically detected events were manually reviewed and reclassified if necessary. All supraventricular runs >5 beats were counted manually to determine the longest episode within the 24-hour interval.

### Echocardiography

As previously reported in detail [7], all transthoracic echocardiograms within Find-AF were performed by study personnel and a comprehensive dataset was prospectively collected. An index specifying left atrial contractility (left atrial volume index as calculated by the ellipsoid formula divided by late diastolic mitral annular velocity as an indicator of atrial contraction = LAVI/a’) was found to be the strongest echocardiographic marker to rule out the presence of PAF and was therefore used for the multivariate analyses in this study.

### Statistics

The prevalence of PAF in patients with and without ESVEA was compared using the Chi-Square-test. Differences in PAC-frequency and the number of beats of the longest SV-run_24 h_ in the PAF vs. the Non-PAF-group were tested using the Mann-Whitney-U-test. The discrimination of ESVEA-parameters between both groups was determined using receiver-operator-characteristic (ROC) curves. We looked for potential influence factors by logistic regression, using age, sex, body-mass-index, systolic blood pressure, hypertension, heart failure and LAVI/a’ as covariates. The clinical parameters used in our analysis had been identified as predictors of incident atrial fibrillation in the Framingham study [16]. PAC/h and SV-run_24 h_ were log-transformed before statistical testing due to skewed distributions. We further included LAVI/a’, the strongest echocardiographic marker for PAF in our study, as we hypothesised that the predictive value of this morphologic indicator of structural atrial remodelling might be distinct from that of ESVEA, an indicator of electrical atrial remodelling. In the case of separation (monotone likelihood), we estimated the effects by Firth’s penalised-likelihood logistic regression following Heinze and Schemper [17]. For this purpose we applied the R package *logistf* [18]. All other analyses were performed by SPSS Statistics version 20. In general, tests were performed as two-sided at 5% significance level.

## Results

**Figure 1:**
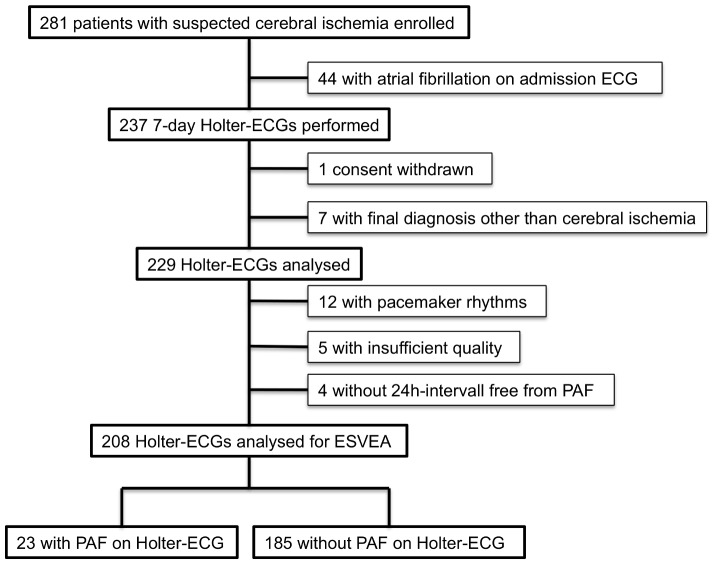
Flow diagram. Flow diagram of patients included in ESVEA analysis within the Find-AF trial. 281 were included in the Find-AF trial, 237 without AF at presentation received 7-day Holter-ECG-monitoring. 229 Holter-ECGs were analysed. ESVEA analysis was possible in 208 cases, 23 of which showed at least one episode of PAF.

229 patients presented in sinus rhythm and therefore underwent 7-day Holter-monitoring within the Find-AF-trial [5]. ESVEA-analysis could not be accomplished in 21 cases (12 pacemaker rhythms, 5 with insufficient quality, 4 without any PAF-free 24-hour interval). The selection of patients for ESVEA-analysis is also shown in the study flow diagram (figure 1). Clinical characteristics are shown in table 1. LAVI/a’ was available in 169 patients of the analysis population.

**Table 1:** Clinical Characteristics.

	No PAF on 7-d-Holter monitoring(n = 185)	PAF on 7-d-Holter monitoring(n = 23)	p-value
**Demographics**			
Female	77 (41.6%)	13 (56.5%)	0.174
Age (y)	^67±13^	^76±12^	0.003
BMI (kg/m^2^)	27.4±5.3	27.6±6.2	0.828
**Risk factors and history**			
SBP (mmHg)	143±22	159±25	0.002
DBP (mmHg)	79±13	81±15	0.361
Heart rate (1/min)	74±15	65±12	0.018
Temperature (°C)	36.7±0.4	36.9±0.5	0.165
History of stroke	27 (14.6%)	5 (21.7%)	0.370
History of TIA	18 (9.7%)	0 (0.0%)	0.118
Heart failure	10 (5.4%)	0 (0.0%)	0.253
Hypertension	130 (70.3%)	20 (87.0%)	0.092
Diabetes	41 (22.2%)	5 (21.7%)	0.963
Smoker	49 (26.5%)	3 (13.0%)	0.106
Hyperlipidemia	58 (31.4%)	12 (52.2%)	0.046
Coronary artery disease	20 (10.8%)	4 (17.4%)	0.352
Peripheral artery disease	5 (2.7%)	1 (4.3%)	0.657
**Laboratory**			
Creatinine (mg/dL)	0.97±0.54	0.95±0.50	0.865
Glucose (mg/dL)	130±54	126±34	0.778
Total cholesterol (mg/dL)	197±46	210±53	0.233
LDL cholesterol (mg/dL)	130±40	138±42	0.400
HDL cholesterol (mg/dL)	51±13	54±20	0.291
Triglycerides (mg/dL)	132±68	112±47	0.177
HbA1c (%)	6.2±1.1	6.5±1.7	0.337
**Stroke severity**			
TIA	67 (36.2%)	1 (4.3%)	
Minor stroke	50 (27.0%)	7 (30.4%)	0.005
Major stroke	68 (36.8%)	15 (65.2%)	
NIHSS	3±4	6±4	<0.001
MRS	2±1	3±1	0.006
ESVEA			
APB/h	4 [1]; [14]	29 [9; 143]	<0.001
SV-run_24h_ (beats)	0 [0; 8]	10 [5]; [21]	<0.001

**Figure 2:**
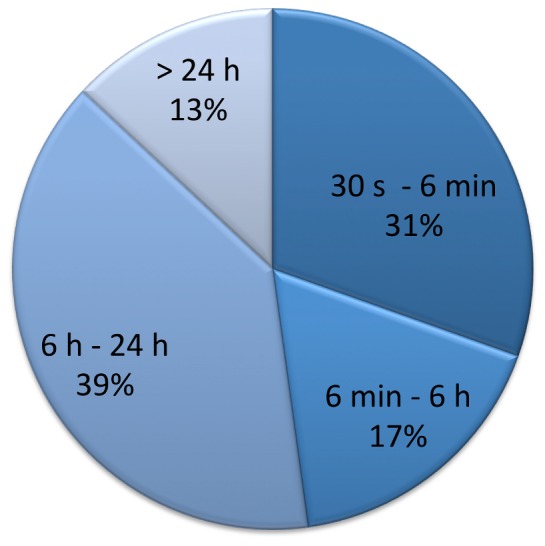
Duration of PAF episodes. Distribution of patients with longest episode of atrial fibrillation in 7-day Holter-monitoring in the respective category. Divisions were chosen based on trials indicating an increased thromboembolic risk above the respective cut-off: 6 min. as shown to increase stroke risk within the ASSERT-trial [24], 6 hours (5.5 hours) as presented by the TRENDS-study investigators [28] and 24 hours as identified within the Italian AT-500 registry [29]. 30 seconds as defined as minimal duration of atrial fibrillation by current AF guidelines [30].

**Figure 3:**
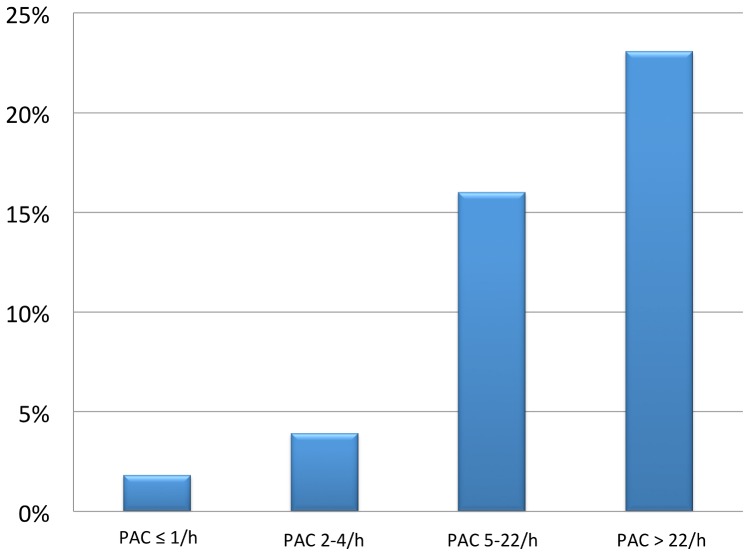
PAF detection on 7-day holter in relation to PACs on 24-hour-Holter. Percentage of patients with paroxysmal atrial fibrillation on 7-day Holter-monitoring across quartiles of PACs/h detected within a 24-hour episode free from PAF.

PAF was detected in 23 of 208 analysed patients (11.1%; Confidence Interval (CI) 6.7%–15.3%). A significant number of patients with PAF showed short bouts, only within the range of minutes (figure 2). The median PAC-frequency was 4/h [IQR 1; 22], the median of SV-run_24 h_ was 5 beats [IQR 0; 9]. PAF was more prevalent in patients with ESVEA: 19.6% (CI 11.5%–27.7%) vs. 2.8% (CI 0.0%–6.2%) for PAC/h >4 vs. ≤4 (p<0.001 chi-square test) (figure 3); 17.0% (CI 10.4%–25.2%) vs. 4.9% (CI 1.1%–9.9%) for longest SV-run_24 h_ >5 vs. ≤5 beats (p = 0.003). Results were similar when PAF-cases were included that were diagnosed clinically until 1-year follow-up: 24.2% (CI 15.8%–33.6%) vs. 3.8% (CI 0.9%–7.9%), p<0.001, for PAC/h >4 vs. ≤4; 20.2% (CI 12.9%–28.0%) vs. 6.9% (CI 2.2–12.2%), p = 0.006, for SV-run_24 h_ >5 vs. ≤5 beats.

Evaluating the cut-off of PAC/h defined by Wallmann et al. [13] yielded comparable results: 16.8% (CI 9.9%–23.8%) vs. 3.4% (CI 0.0%–7.6%) for PAC/h >3 vs. ≤3. Applying the cut-offs for PAC/h and longest SV-run_24 h_ as defined for initially healthy subjects by Binici et al. [15] showed slightly different PAF rates: 24.4% (CI 11.1%–38.5%) vs. 7.4% (CI 3.6%–11.7%), p = 0.001, for PAC/h >30 vs. ≤30 and 37.5% (CI 13.3%–64.3%) vs. 8.9% (CI 5.3%–12.9%), p<0.001, for SV-run_24 h_ >20 vs. ≤20 beats.

Patients with PAF showed more supraventricular ectopic activity: PAC/h = 29 [IQR 9; 143] vs. 4 [1]; [14] and SV-run_24 h_ = 10 [5]; [21] vs. 0 [0; 8] beats (both p<0.001).

**Figure 4:**
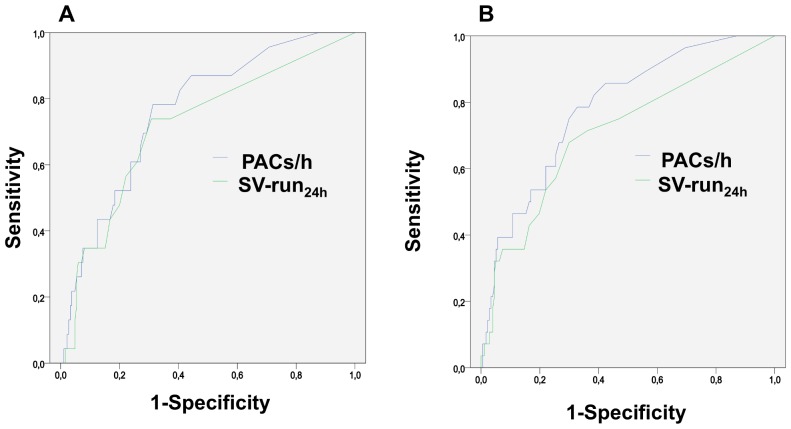
ROC-curves – ESVEA as a predictor of PAF. ROC-curves for PAC/h (blue) and SV-run_24 h_ (green) to detect paroxysmal atrial fibrillation A) in baseline 7-day Holter-monitoring only or B) total PAF after baseline 7-day Holter-monitoring and clinical follow-up (up to 1 year).

Both parameters, PAC/h and SV-run_24 h_, discriminated between the PAF and the non-PAF group (area under the ROC curve = 0.763 [CI 0.667–0.858] and 0.716 [0.600–0.832]) (figure 4).

At the optimal cut-off-point (maximal Youden-index), sensitivity, specificity, negative and positive predictive value were 78.3%, 68.6%, 96% and 24% for PAC/h (8/h) and 73.9%, 69.2%, 96% and 23% for SV-run_24 h_ (6 beats). The evaluated cut-offs by Binici et al. [15] slightly improved specificity at the cost of greatly impaired sensitivity. Sensitivity, specificity, positive and negative predictive value were 47.8%, 81.6%, 24.4% and 92.6% for PAC/h and 26.1%, 94.6%, 37.5% and 91.1% for longest SV-run_24 h_.

The predictive value of the ESVEA parameters was confirmed by repeating the analyses, taking into account only cases whose ESVEA parameters had been determined in an ECG-interval before first documentation of PAF. The revised median frequency of PAC remained 4/h and PAF prevalence remained significantly higher in those with PAC/h >4 (11.8%; CI 5.4%–18.7% vs. 1.9%; CI 0.0%–4.8%, p = 0.005). The revised median for longest SV-run_24 h_, however, turned out to be 0 with the smaller number of PAF-patients and we did not apply this cut-off to calculate the differences in PAF prevalence. ESVEA rates remained higher in the PAF vs. the non-PAF group: the median was 11 (IQR 5/106) vs. 4 (IQR 1/15) for PAC/h and 12 (IQR 5/12) vs. 0 (IQR 0/8) for the longest SV-run_24 h_. Both markers still discriminated between the PAF and the non-PAF group: The area under the ROC curve was 0.686 (CI 0.540–0.832), p = 0.038 for PAC/h and 0.733 (CI 0.575–0.891), p = 0.01 for the longest SV-run_24 h_.

In multivariate analyses using age, sex, body-mass index, systolic blood pressure, hypertension and heart failure as covariates, log(PAC/h) (p = 0.002) and log(SV-run_24 h_) (p = 0.007) were independently associated with PAF (table S1A in file S1). The same was observed in a different model based on baseline differences between the groups (table S1B in file S1) as well as in a combination of both models (table S2 in file S1). Corrected for other significant predictors, patients with PAC/h >4 had an odds ratio of 5.7 (p = 0.011) and those with SV-run_24 h_ >5 beats of 3.1 (p = 0.054) of having PAF (table S3 in file S1). Importantly, both ESVEA parameters maintained significance when LAVI/a’, the strongest echocardiographic predictor, was entered into the model (table S4A in file S1), with an adjusted odds ratio of 4.4 (p = 0.048) for PAC/h and 3.1 (p = 0.109) for SV-run_24 h_ above the median (table S4B in file S1).

PAC/h ≤4+ normal LAVI/a’-index (i.e. <2.3) excluded presence of PAF (0/55 = 0.0%; CI 0.0%–6.5%), whereas PAF-rates were high in patients with PAC/h >4 and LAVI/a’ ≥2.3 (14/47 = 29.8%; CI 18.7%–44.0%).

Among those patients for whom LAVI/a’ was available, 8 (4.7%) experienced a recurrent stroke during follow-up. Seven of those (87.5%) had initially shown a pathologic LAVI/a’-index, whereas only 3 (37.5%) had had frequent PAC.

## Discussion

Our results imply that patients with ESVEA on 24-hour Holter-ECG show significantly higher PAF-rates within the entire, prolonged monitoring spell. Both parameters, frequent PAC and longest SV-run_24 h_, are significantly and independently associated with underlying PAF. When combining PAC/h, a marker of atrial electric remodelling, with LAVI/a’, an echocardiographic marker of structural atrial remodelling, PAF was literally ruled out in patients with normal results.

### Association of ESVEA and PAF

Several investigators found that episodes of AF are often initiated by premature atrial complexes [19]–[21]. Some previous publications have examined the association of excessive supraventricular ectopic activity and the finding of atrial fibrillation. Healthy subjects with high rates of PAC or longer SV-runs on 24-hour Holter were shown to be at a higher risk of developing atrial fibrillation or experiencing cerebral ischemia [14]–[15]. Retrospective analyses of Holter-ECGs recorded from patients after ischemic strokes showed a correlation of frequent PAC and finding of PAF [10]–[11]. Wallmann et al. analysed frequent PAC in patients with acute cerebral ischemia as an indicator of underlying PAF. Detection rates on delayed extended monitoring were significantly higher in those with more than approximately 3 PAC/h (70 PAC/d) on an initial 24-hour Holter-ECG [12]–[13]. Our data are in good agreement with Wallmann’s findings: In our study population, those with >4 PAC/h (median) in a 24-hour ECG-recording were found to have significantly more PAF within a prolonged (7-day) period. Our cut-off lies within close range of that defined by Wallmann et al. (>4 PAC/h vs. >3 PAC/h). In addition to PAC/h, we identified the longest supraventricular run as another valid factor associated with underlying PAF in patients with recent stroke or TIA. Evaluating ESVEA cut-offs previously defined for initially healthy patients by Binici et al. [15], the applied thresholds appear to be too strict for patients with cerebral ischemia, as sensitivity is greatly reduced, while gaining only moderately increased specificity. Altogether, PAC/h appeared to show a more significant association with PAF and the combination with the longest SV-run_24 h_ did not improve the results.

### Electrocardiographic and Echocardiographic Markers Help to Identify Patients at High Risk of Underlying PAF

We expected electrocardiographic/echocardiographic markers, both indicators of atrial pathologies, to be considerably more specific surrogates of atrial fibrillation compared to unspecific clinical parameters currently taken into account [22]–[23].

Multivariate analysis containing both PAC/h and LAVI/a’ confirmed that both parameters are independently associated with PAF, possibly because structural and electrical remodelling occur separately, depending on the patient’s individual AF-aetiology. Interestingly, PAF was literally ruled out in those who showed neither high rates of PAC on 24-hour Holter-ECG, nor signs of structural remodelling in echocardiography. Therefore it makes sense to combine both indicators to rule out atrial fibrillation in those without structural or electrical atrial alterations.

### ESVEA and LAVI/a’ as Prognostic Markers?

Within the Find-AF-trial we also reviewed cardiovascular events in up to one year of follow-up. Interestingly, our data suggest LAVI/a’ may be a risk factor for recurrent strokes, while ESVEA, albeit similarly a surrogate for PAF in our cohort, does not appear to hold predictive value here. This might indicate that left atrial mechanical dysfunction per se is a risk factor for recurrent thromboembolism, while electrical instability is of lesser importance. However, this analysis is limited by small numbers and can be considered as hypothesis-generating at best.

### Clinical Relevance and Perspective

Compared to other common aetiologies of cerebral ischemia, paroxysmal AF is difficult to diagnose. Apart from being asymptomatic in a high percentage of cases [2], AF-bursts are often of short duration (while even short spells significantly increase stroke risk [24]) and furthermore they often appear in clusters (bearing the possibility of long periods free from the arrhythmia). Therefore PAF-detection-rates significantly increase by prolonging the monitoring spell.

Detection of PAF, however, is highly relevant, because oral anticoagulation has been shown to reduce recurrent ischemic events significantly, along with improving long-term prognosis [25]–[26]. Throughout the last years, methods for AF-detection have constantly improved [6]. New developments include implantable loop-recorders, that allow significantly longer monitoring periods and therefore offer the opportunity for presumably more sensitive AF-detection. While externally applied devices are generally cumbersome for patients and often bear a high workload for investigators, implanted devices require invasive procedures for implantation and explanation and all procedures are considerably expensive, limiting a wide spread utilisation in clinical practice.

This calls for valid and easily acquired markers of underlying PAF in patients with recent cerebral ischemia to increase the pre-test probability in order to focus elaborate extended monitoring procedures on patients at a high risk of underlying AF. Several factors associated with AF have been identified [16] and risk scores have been published [22]–[23], the latter leaving room for further improvement [27]. Most importantly, we would like to point out, that many of the markers currently taken into account (e.g. age, gender, hypertension, cardiovascular disease, BMI) are very unspecific of PAF, rather indicating cardiovascular disease in general.

In search of more specific markers, we wanted to focus on indicators of atrial pathologies as supposedly better surrogates for atrial fibrillation. We used electrocardiographic parameters indicating electrical remodelling (frequent PAC and longer duration of short SV-runs summarised as ESVEA) as well as echocardiographic markers of structural remodelling of the atrium (LAVI/a’), both processes known to be associated with atrial fibrillation [9]. Importantly, both these markers are easily attained by standard clinical procedures performed after ischemic stroke and may be used complementary, depending on local availability.

Based on our findings, electrocardiographic and echocardiographic markers could help future development/improvement of predictive scores for underlying PAF in patients with acute cerebral ischemia. Their aim will be to select those with a high probability of PAF for extended ECG-monitoring.

### Limitations

ESVEA was analysed retrospectively and were not a primary goal of the study. Furthermore, the 24-hour Holter-interval used for ESVEA-analysis was taken from the same, prolonged spell used for PAF detection. In our main analysis we investigated the association of ESVEA with PAF occurring both before and after ESVEA analysis as the best indicator of truly underlying PAF, which per se is not fully identical to predicting future detection (and possibly new incidence) of PAF. Although we confirmed the predictive value of ESVEA after excluding all cases of PAF that had occurred before ESVEA analysis, this confirmatory analysis is limited by the reduced number of PAF cases.

### Summary

Electrocardiographic markers of atrial electric remodelling, PACs/h and longest SV-run_24 h_, could be useful tools to identify patients with recent cerebral ischemia at high risk of underlying paroxysmal atrial fibrillation. In our cohort of patients with acute cerebral ischemia, PAF was ruled out in those without ESVEA on 24-hour Holter-ECG and with normal LAVI/a’, an echocardiographic marker of structural remodelling.

## Supporting Information

File S1 **Multiple Binary Regression Models. Table S1A.** Co-variables based on a predictive model for incident atrial fibrillation (Schnabel et al. [16]). All co-variables forced into the model. **Table S1B.** Co-variables based on significant baseline differences between groups. All co-variables forced into the model. **Table S2.** Co-variables based on significant contributors in models in tables S1A & S1B. Stepwise inclusion due to number of co-variables for model S2/1, forced inclusion for models S2/2 and 3 to keep the base model and examine gain by adding ESVEA parameters. **Table S3.** Based on models in table S2, ESVEA parameters were entered as dichotomised variable (above vs. below median). **Table S4A.** Based on models in table S2, LAVI/a’ was added as the strongest echocardiographic predictor of PAF. **Table S4B.** Based on models in table S2, LAVI/a’ was added as the strongest echocardiographic predictor of PAF.
(DOCX)

## References

[1] Kirchhof P, Auricchio A, Bax J, Crijns H, Camm J, et al (2007) Outcome parameters for trials in atrial fibrillation: executive summary. Recommendations from a consensus conference organized by the German Atrial Fibrillation Competence NETwork (AFNET) and the European Heart Rhythm Association (EHRA). Eur Heart J 28: 2803–2817. 17897924 10.1093/eurheartj/ehm358

[2] Page RL, Wilkinson WE, Clair WK, McCarthy EA, Pritchett EL (1994) Asymptomatic arrhythmias in patients with symptomatic paroxysmal atrial fibrillation and paroxysmal supraventricular tachycardia. Circulation 89: 224–227. 8281651 10.1161/01.cir.89.1.224

[3] Liao J, Khalid Z, Scallan C, Morillo C, O’Donnell M (2007) Noninvasive cardiac monitoring for detecting paroxysmal atrial fibrillation or flutter after acute ischemic stroke: a systematic review. Stroke 38: 2935–2940. 17901394 10.1161/STROKEAHA.106.478685

[4] Seet RC, Friedman PA, Rabinstein AA (2011) Prolonged rhythm monitoring for the detection of occult paroxysmal atrial fibrillation in ischemic stroke of unknown cause. Circulation 26 124: 477–486. 10.1161/CIRCULATIONAHA.111.029801 21788600

[5] Stahrenberg R, Weber-Krüger M, Seegers J, Edelmann F, Lahno R, et al (2010) Enhanced detection of paroxysmal atrial fibrillation by early and prolonged continuous holter monitoring in patients with cerebral ischemia presenting in sinus rhythm. Stroke 41: 2884–2888. 20966415 10.1161/STROKEAHA.110.591958

[6] Mittal S, Movsowitz C, Steinberg JS (2011) Ambulatory external electrocardiographic monitoring: focus on atrial fibrillation. J Am Coll Cardiol 58: 1741–1749. 21996384 10.1016/j.jacc.2011.07.026

[7] Stahrenberg R, Edelmann F, Haase B, Lahno R, Seegers J, et al (2011) Transthoracic echocardiography to rule out paroxysmal atrial fibrillation as a cause of stroke or transient ischemic attack. Stroke 42: 3643–3645. 21998056 10.1161/STROKEAHA.111.632836

[8] Wachter R, Lahno R, Haase B, Weber-Krüger M, Seegers J, et al (2012) Natriuretic peptides for the detection of paroxysmal atrial fibrillation in patients with cerebral ischemia – the Find-AF study. PLOS one 7: e34351. 22509292 10.1371/journal.pone.0034351 PMC3324530

[9] Schotten U, Verheule S, Kirchhof P, Goette A (2011) Pathophysiological mechanisms of atrial fibrillation: a translational appraisal. Physiol Rev 91: 265–325. Review. Erratum in: Physiol Rev. 2011 91: 1533. 10.1152/physrev.00031.2009 21248168

[10] Koudstaal PJ, van Gijn J, Klootwijk AP, van der Meche FG, Kappelle LJ (1986) Holter monitoring in patients with transient and focal ischemic attacks of the brain. Stroke 17: 192–195. 2938308 10.1161/01.str.17.2.192

[11] Gaillard N, Deltour S, Vilotijevic B, Hornych A, Crozier S, et al (2010) Detection of paroxysmal atrial fibrillation with transtelephonic EKG in TIA or stroke patients. Neurology 74: 1666–1670. 20498434 10.1212/WNL.0b013e3181e0427e

[12] Wallmann D, Tüller D, Kucher N, Fuhrer J, Arnold M, et al (2003) Frequent atrial premature contractions as a surrogate marker for paroxysmal atrial fibrillation in patients with acute ischaemic stroke. Heart 89: 1247–1248. 12975433 10.1136/heart.89.10.1247 PMC1767912

[13] Wallmann D, Tüller D, Wustmann K, Meier P, Isenegger J, et al (2007) Frequent atrial premature beats predict paroxysmal atrial fibrillation in stroke patients: an opportunity for a new diagnostic strategy. Stroke 38: 2292–2294. 17585079 10.1161/STROKEAHA.107.485110

[14] Engström G, Hedblad B, Juul-Möller S, Tydén P, Janzon L (2000) Cardiac arrhythmias and stroke: increased risk in men with high frequency of atrial ectopic beats. Stroke 31: 2925–2929. 11108750 10.1161/01.str.31.12.2925

[15] Binici Z, Intzilakis T, Nielsen OW, Kober L, Sajadieh A (2010) Excessive Supraventricular ectopic activity and increased risk of atrial fibrillation and stroke. Circulation 121: 1904–1911. 20404258 10.1161/CIRCULATIONAHA.109.874982

[16] Schnabel RB, Sullivan LM, Levy D, Pencina MJ, Massaro JM, et al (2009) Development of a risk score for atrial fibrillation (Framingham Heart Study): a community-based cohort study. Lancet 373: 739–45. 19249635 10.1016/S0140-6736(09)60443-8 PMC2764235

[17] Heinze G, Schemper M (2002) A solution to the problem of separation in logistic regression. Stat Med 21: 2409–2419. 12210625 10.1002/sim.1047

[18] R Development Core Team. R: A language and environment for statistical computing. R Foundation for Statistical Computing 2011, Vienna, Austria. ISBN 3–900051–07–0, URL http://www.R-project.org/.

[19] Killip T, Gault JH (1965) Mode of onset of atrial fibrillation in man. Am Heart J 70: 172–179. 14328002 10.1016/0002-8703(65)90064-5

[20] Bennett MA, Pentecost BL (1970) The pattern of onset and spontaneous cessation of atrial fibrillation in man. Circulation 41: 981–988. 5482912 10.1161/01.cir.41.6.981

[21] Capucci A, Santarelli A, Boriani G, Magnani B (1992) Atrial premature beats coupling interval determines lone paroxysmal atrial fibrillation onset. Int J Cardiol 36: 87–93. 1385338 10.1016/0167-5273(92)90112-g

[22] Malik S, Hicks WJ, Schultz L, Penstone P, Gardner J, et al (2011) Development of a scoring system for atrial fibrillation in acute stroke and transient ischemic attack patients: the LADS scoring system. J Neurol Sci 301: 27–30. 21130468 10.1016/j.jns.2010.11.011

[23] Suissa L, Bertora D, Lachaud S, Mahagne MH (2009) Score for the targeting of atrial fibrillation (STAF): a new approach to the detection of atrial fibrillation in the secondary prevention of ischemic stroke. Stroke 40: 2866–2868. 19461041 10.1161/STROKEAHA.109.552679

[24] Healey JS, Connolly SJ, Gold MR, Israel CW, Van Gelder IC, et al (2012) Subclinical atrial fibrillation and the risk of stroke. N Engl J Med 366: 120–129. 22236222 10.1056/NEJMoa1105575

[25] Hart RG, Halperin JL, Pearce LA, Anderson DC, Kronmal RA, et al (2003) Stroke Prevention in Atrial Fibrillation Investigators. Lessons from the Stroke Prevention in Atrial Fibrillation trials. Ann Intern Med 138: 831–838. 12755555 10.7326/0003-4819-138-10-200305200-00011

[26] Crystal E, Connolly SJ (2004) Role of oral anticoagulation in management of atrial fibrillation. Heart 90: 813–817. 15201261 10.1136/hrt.2003.021642 PMC1768341

[27] Stahrenberg R, Wachter R, Gröschel K (2010) A risk score to predict future atrial fibrillation derived from patients with stroke initially presenting with atrial fibrillation? Stroke 41: e169. 20133915 10.1161/STROKEAHA.109.573675

[28] Glotzer TV, Daoud EG, Wyse DG, Singer DE, Ezekowitz MD, et al (2009) The relationship between daily atrial tachyarrhythmia burden from implantable device diagnostics and stroke risk: the TRENDS study. Circ Arrhythm Electrophysiol. 2: 474–480. 10.1161/CIRCEP.109.849638 19843914

[29] Capucci A, Santini M, Padeletti L, Gulizia M, Botto G, et al (2005) Monitored atrial fibrillation duration predicts arterial embolic events in patients suffering from bradycardia and atrial fibrillation implanted with antitachycardia pacemakers. J Am Coll Cardiol. 46: 1913–1920. 10.1016/j.jacc.2005.07.044 16286180

[30] Camm AJ, Kirchhof P, Lip GY, Schotten U, Savelieva I, et al (2010) Guidelines for the management of atrial fibrillation: the Task Force for the Management of Atrial Fibrillation of the European Society of Cardiology (ESC). Eur Heart J. 31: 2369–2429. 10.1093/eurheartj/ehq278 20802247

