# Supplementary material for: Excessive Supraventricular Ectopic Activity Is Indicative of Paroxysmal Atrial Fibrillation in Patients with Cerebral Ischemia
Source: PLoS One. 2013 Jun 28;8(6):e67602. doi: 10.1371/journal.pone.0067602 (PMC3695922; doi:10.1371/journal.pone.0067602)
Supplement: File S1 — Multiple Binary Regression Models. Table S1A. Co-variables based on a predictive model for incident atrial fibrillation (Schnabel et al. [16]). All co-variables forced into the model. Table S1B. Co-variables based on significant baseline differences between groups. All co-variables forced into the model. Table S2. Co-variables based on significant contributors in models in tables S1A & S1B. Stepwise inclusion due to number of co-variables for model S2/1, forced inclusion for models S2/2 and 3 to keep the base model and examine gain by adding ESVEA parameters. Table S3. Based on models in table S2, ESVEA parameters were entered as dichotomised variable (above vs. below median). Table S4A. Based on models in table S2, LAVI/a’ was added as the strongest echocardiographic predictor of PAF. Table S4B. Based on models in table S2, LAVI/a’ was added as the strongest echocardiographic predictor of PAF. (DOCX) [file pone.0067602.s001.docx]

**File S1 - Supporting Information:**

**Multivariate Binary Logistic Regression models**

**Table S1A:**

Multivariate binary logistic regression models. Co-variables based on a predictive model for incident atrial fibrillation (Schnabel et al. [16]). All co-variables forced into the model.

|  | Model S1A/1 | | Model S1A/2 | | Model S1A/3 | |
| --- | --- | --- | --- | --- | --- | --- |
|  | OR | p-value | OR | p-value | OR | p-value |
| Systolic blood pressure | 1.02 | 0.017 | 1.02 | 0.031 | 1.03 | 0.010 |
| Heart failure | 0.38 | 0.465 | 0.44 | 0.554 | 0.36 | 0.435 |
| Gender | 1.76 | 0.236 | 2.12 | 0.128 | 1.37 | 0.521 |
| Age | 1.05 | 0.027 | 1.02 | 0.402 | 1.03 | 0.202 |
| BMI | 1.02 | 0.670 | 1.02 | 0.547 | 1.03 | 0.487 |
| Lg (APCs/h) |  |  | 2.86 | 0.002 |  |  |
| Lg (SV-run_24h_) |  |  |  |  | 3.45 | 0.007 |

**Table S1B:**

Multivariate binary logistic regression models. Co-variables based on significant baseline differences between groups. All co-variables forced into the model.

|  | Model S1B/1 | | Model S1B/2 | | Model S1B/3 | |
| --- | --- | --- | --- | --- | --- | --- |
|  | OR | p-value | OR | p-value | OR | p-value |
| Systolic blood pressure |  |  | 1.02 | 0.030 | 1.03 | 0.012 |
| Age |  |  | 1.03 | 0.267 | 1.04 | 0.131 |
| Heart rate |  |  | 0.99 | 0.516 | 0.99 | 0.459 |
| Hyperlipidemia |  |  | 2.98 | 0.038 | 3.14 | 0.028 |
| NIH-SS |  |  | 1.15 | 0.011 | 1.15 | 0.010 |
| Lg (APCs/h) |  |  | 2.70 | 0.006 |  |  |
| Lg (SV-run_24h_) |  |  |  |  | 3.00 | 0.017 |

**Table S2:**

Multivariate binary logistic regression models. Co-variables based on significant contributors in models in table 1. Stepwise inclusion due to number of co-variables for model 1, forced inclusion for models 2 and 3 to keep the base model and examine gain by adding ESVEA parameters.

|  | Model S2/1 | | Model S2/2 | | Model S2/3 | |
| --- | --- | --- | --- | --- | --- | --- |
|  | OR | p-value | OR | p-value | OR | p-value |
| Systolic blood pressure | 1.02 | 0.035 | 1.02 | 0.035 | 1.03 | 0.015 |
| Age | 1.05 | 0.047 | 1.03 | 0.244 | 1.04 | 0.111 |
| Hyperlipidemia | 3.82 | 0.013 | 3.23 | 0.022 | 3.31 | 0.020 |
| NIH-SS | 1.21 | 0.003 | 1.15 | 0.011 | 1.14 | 0.012 |
| Lg (APCs/h) |  |  | 2.71 | 0.005 |  |  |
| Lg (SV-run_24h_) |  |  |  |  | 3.02 | 0.016 |

Variables not entering the equation for model 1 in stepwise inclusion procedure:
gender, heart rate, heart failure, BMI

**Table S3:**

Multivariate binary logistic regression models. Based on models in table 2, ESVEA parameters were entered as dichotomised variable (above vs. below median).

|  | Model S3/1 | | Model S3/2 | |
| --- | --- | --- | --- | --- |
|  | OR | p-value | OR | p-value |
| Systolic blood pressure | 1.02 | 0.031 | 1.03 | 0.013 |
| Age | 1.04 | 0.198 | 1.04 | 0.107 |
| Hyperlipidemia | 3.57 | 0.013 | 3.33 | 0.018 |
| NIH-SS | 1.13 | 0.018 | 1.14 | 0.014 |
| APCs/h > median | 5.71 | 0.011 |  |  |
| SV-run_24h_ > median |  |  | 3.12 | 0.054 |

**Table S4A:** Multivariate binary logistic regression models. Based on models in table 2, LAVI/a´ was added as the strongest echocardiographic predictor of PAF.

|  | Model S4A/1 | | Model S4A/2 | |
| --- | --- | --- | --- | --- |
|  | OR | p-value | OR | p-value |
| Systolic blood pressure | 1.02 | 0.114 | 1.02 | 0.042 |
| Age | 1.02 | 0.572 | 1.04 | 0.251 |
| Hyperlipidemia | 6.82 | 0.005 | 5.71 | 0.009 |
| NIH-SS | 1.24 | 0.008 | 1.22 | 0.014 |
| LAVI/a´ | 1.37 | 0.023 | 1.41 | 0.014 |
| Lg (APCs/h) | 3.49 | 0.006 |  |  |
| Lg (SV-run_24h_) |  |  | 3.40 | 0.029 |

**Table S4B:** Multivariate binary logistic regression models. Based on models in table 2, LAVI/a´ was added as the strongest echocardiographic predictor of PAF.

|  | Model S4B/1 | | Model S4B/2 | |
| --- | --- | --- | --- | --- |
|  | OR | p-value | OR | p-value |
| Systolic blood pressure | 1.02 | 0.130 | 1.02 | 0.045 |
| Age | 1.04 | 0.289 | 1.04 | 0.226 |
| Hyperlipidemia | 6.60 | 0.005 | 5.71 | 0.008 |
| NIH-SS | 1.21 | 0.015 | 1.21 | 0.014 |
| LAVI/a´ | 1.35 | 0.025 | 1.39 | 0.013 |
| APCs/h > median | 4.38 | 0.048 |  |  |
| SV-run_24h_ > median |  |  | 3.13 | 0.109 |
